# Factors confounding fluorescein-guided malignant glioma resections: edema bulk flow, dose, timing, and now: imaging hardware?

**DOI:** 10.1007/s00701-015-2655-6

**Published:** 2015-12-07

**Authors:** Walter Stummer

**Affiliations:** Neurochirurgische Klinik, Universitätsklinikum Münster, Albert-Schweitzer-Campus 1, Gebäude A1, 48149 Münster, Germany

Dear Sir,

I thank you for the opportunity to comment a letter to Acta Neurochirurgica by Dr. Bongetta and coworkers regarding low-price tailored hardware for fluorescein imaging during the resection of malignant gliomas. This group advocates the use of simple blue light available with commercially available endoscopy systems for excitation and a commercially available yellow optical filter fit to the microscope oculars for observation of fluorescence. The argumentation on their side is that more affordable systems would help those surgeons with low budgets and lack of assets to have access to fluorescence-guided resections.

Similar solutions, on a side note, are also available for ALA porphyrin imaging, using filters with exactly the same specifications incorporated into currently available Zeiss or Leica microscopes (D-Light, Storz, Tuttlingen, Germany). In fact, we ourselves began with handheld filters and a xenon light source filtered for blue-violet light (375–440 nm) using identical filters (unpublished data). Unfortunately, even though these systems are available, their versatility during surgery is restricted. The full potential of fluorescence imaging comes from the integration into the operating microscope, which, as the authors correctly point out, is also the most expensive solution. So given a confirmed usefulness of a particular fluorophore for fluorescence-guided resections, cheaper solutions might be available and the authors are correct in pointing this out.

However, the authors’ proposal also raises new concerns regarding fluorescein-guided resections, and that is the possible influence of the hardware on what the surgeon sees. Investigators of fluorescein are currently using multitudes of different doses and timing, between 3 and 20 mg/kg, administered by either acute injections, injections before opening of the dura, or injections after induction of anesthesia [1–3, 5–9].

Personally, I would prefer (if at all) 3–4 mg fluorescein administered after induction of anesthesia, according to our experience with the method [10, 11] using the Zeiss Yellow 560 filter module. This particular mode of use was defined by Ascerbi et al. [1, 2] using the same Zeiss filter system, which has (proprietary) transmission characteristics, and was an important impetus for currently reconsidering the use of fluorescein in malignant glioma and vascular surgery, the former use first described in 1948 [6]. To this end, it remains to be established whether the low-cost solution by Bongetta et al. shows the same type of tissue delineation that appears to be observed using the Yellow 560 filter and whether earlier observations can simply be extrapolated to their particular fluorescein imaging solution.

A different hardware solution with differing light source and detection filter specifications provides new confounders to a technique, which is already beset with multiple variables.

Unfortunately, the basic problems involving fluorescein (a marker of blood–brain barrier integrity) [12] for fluorescence-guided resections remain under investigated. If given too acutely, fluorescein concentrations will be high in all perfused tissues and vessels. In this situation, if tissue is perturbed by surgery, there is unspecific extravasation of fluorescein unrelated to tumor, especially along the resection margins. If, conversely, fluorescein is given with too long of a delay to the actual resection, there are legitimate concerns about where the fluorescein is going in the brain, whereas the problems with unspecific extravasation at the resection margins remains remain. Tumor-related edema in the brain, which was intensely investigated in the 1970s and 1980s, will transverse tissue at a speed exceeding 2 mm per hour [4] in patients. With 2 to 3 h between administration of fluorescein and interrogation of tissue, this marker will unspecifically have traversed white matter up to 5 mm, a worrisome thought in the context of an intra-operative tumor marker.

These confounders have to be kept in mind for fluorophores that do not have a specific tumor–fluorophore interaction, as is the case for fluorescein [3]. Thus, while I agree with Bongetta et al. that less-costly hardware solutions would be helpful for dissemination of many techniques in neurosurgery, the biological or theoretical basis for any technique needs to be well defined and the solutions should not provide an additional confounder in an already-complex environment.

Sincerely,

Walter Stummer
